# Discriminating Between Marijuana and Alcohol Gait Impairments Using Tile CNN With TICA Pooling

**DOI:** 10.1109/OJEMB.2025.3607556

**Published:** 2025-09-09

**Authors:** Ruojun Li, Samuel Chibuoyim Uche, Emmanuel Agu, Kristin Grimone, Debra S. Herman, Jane Metrik, Ana M. Abrantes, Michael D. Stein

**Affiliations:** Worcester Polytechnic Institute Worcester MA 01609 USA; Butler Hospital Providence RI 02906 USA; Brown University Providence RI 02912 USA; Boston University Boston MA 02215 USA

**Keywords:** Accelerometer, alcohol impairment, deep learning, gyroscope, marijuana impairment

## Abstract

*Goal:* To investigate whether machine learning analyses of smartphone sensor data can discriminate whether a subject consumed alcohol or marijuana from their gait. *Methods:* Using first-of-a-kind impaired gait datasets, we propose *MariaGait*, a novel deep learning approach to distinguish between marijuana and alcohol impairment. Subjects' time-series smartphone accelerometer and gyroscope sensor gait data are first encoded into Gramian Angular Field (GAF) images that are then classified using a tiled Convolutional Neural Network (CNN) with TICA pooling. To mitigate the insufficiency of positively labeled alcohol and marijuana instances, the tiled CNN was pre-trained on sober gait samples that were more abundant. *Results:* *MariaGait* achieved an accuracy of 94.61%, F1 score of 88.61%, and 94.33% ROC AUC score in classifying whether the subject consumed alcohol or marijuana, outperforming baseline models including Multi-Layer Perceptron (MLP), Long Short Term Memory (LSTM), Multi-head CNN and Multi-head LSTM, Random Forest and Support Vector Machines (SVM)). *Conclusions:* Our results demonstrate that *MariaGait* could be a practical, non-invasive approach to determine which substance a subject is impaired by from their gait.

## I. Introduction

**MOTIVATION:** Alcohol and marijuana are widely consumed substances that impair judgment, coordination, reaction time, and motor skills, significantly increasing the risk of impaired driving [1], [2]. In 2023, 61.8 million individuals 12 years and older in the U.S. used marijuana [3], while alcohol consumption affected 1.34 billion people globally in 2020 [4]. In the U.S., alcohol consumption rose from 6.3% to 9.6% in 2021 [5], with 134.7 million people reporting alcohol use, 61.4 million of whom engaged in binge drinking [3]. Driving under the influence (DUI) incidents contribute significantly to traffic fatalities, with alcohol-impaired driving involved in one in three cases [6], and marijuana-related driving incidents increasing five- to tenfold since 2000 [7]. Emergency department visits for marijuana-related motor injuries surged by 475.3% from 2010 to 2021 [8]. These trends highlight the urgent need for effective impairment detection to improve road safety and assist law enforcement in distinguishing alcohol from marijuana impairment due to varying legal repercussions.

Alcohol impairment is measured by Blood Alcohol Concentration (BAC), while marijuana impairment correlates with $\Delta ^{9}$-*tetrahydrocannabinol* (THC) levels [9]. A BAC of $\geq$0.08% is the legal DUI threshold, detectable via breathalyzers, though these devices require purchase and active use. THC Breath Analyzers (THCBA) remain experimental and lack portability [10]. The SoToxa test [11], involving oral fluid analysis, can be used for roadside detection of marijuana impairment but is not suitable for continuous monitoring [12]. Drug Recognition Experts (DREs) offer more accurate impairment assessments [13], but the process is costly, invasive, and requires cooperation [14]. A non-invasive, portable solution is needed to determine whether impaired subjects consumed alcohol or marijuana especially because the legal penalties for these substances vary with the jurisdiction. Beyond DUI enforcement, the ability to distinguish between marijuana and alcohol impairment has significant implications for clinical monitoring, workplace safety, healthcare and substance abuse treatment. For instance, studies have shown that alcohol (which impairs the cerebellum) and marijuana (which impairs the cerebellum and basal ganglia) impair gait and balance in distinct ways, affecting the risk profiles for falls, motor performance, and cognitive processing [15], [16]. In workplace settings, substance-specific detection can be used to support the enforcement of relevant substance-related policies, to tailor risk assessment and interventions, and support legal fairness [17], [18], [19]. In healthcare, such a capability may inform passive gait assessment for neurocognitive impairment screening, substance use disorder monitoring and personalized intervention strategies, and provide information required by medical professionals to make decisions during impairment-related emergencies [19], [20], [21].

Machine learning (ML) has been applied to impairment detection via facial analysis [22], [23], [24]. However, facial analysis is sensitive to lighting and camera position, limiting its generalizability. Sensor-based methods have explored breath analyses via sensors embedded in steering wheels, ultrasonic, accelerometer, and vibration sensors [25], but these approaches need to be installed and do not facilitate continuous monitoring. Floor-mounted, pressure-based systems detect alcohol-induced gait impairments [26] but lack portability for continuous monitoring. Gait is a reliable biomarker of substance-induced impairment [27]. With the widespread adoption of smartphones, data collected from their built-in accelerometers and gyroscopes combined with machine learning analyses offer a non-invasive, cost-effective approach for real-time gait analysis. Prior studies have explored ML methods to estimate BAC from smartphone gait sensor data [28], [29] or detect marijuana impairment [30], [31]. However, most of these works were separate studies that focused on detecting either impairment generally or estimating substance quantity, rather than discriminating between whether alcohol vs. marijuana was consumed based on their impaired gait data. Furthermore, no prior study have explored image-based encodings such as Gramian Angular Fields (GAF) to transform time-series sensor data, enabling them to be classified by state-of-the-art CNNs, for the purpose of discriminating marijuana–alcohol substance impairment. A few works have explored classification of GAF encodings to detect impairment by alcohol [27] or marijuana [32]. Our work fills this gap, being the first to discriminate between alcohol- and marijuana-induced impairment from smartphone-captured gait sensor data encoded as GAF images.

*Specific Problem:* This study investigates whether ML methods on smartphone sensor gait data can accurately distinguish alcohol- versus marijuana-induced impairment.

*Challenges:* Discriminating between alcohol- and marijuana-induced gait impairment is challenging because some of their effects on coordination and balance are similar. Variability in individual responses due to metabolism, tolerance, and body composition can confuse ML classifiers. Publicly available impaired smartphone gait data is limited, and datasets are often imbalanced, which presents a challenge to ML classification. Additionally, variations in smartphone hardware, sensor accuracy, and placement can impact gait data quality.

**Fig. 1.**
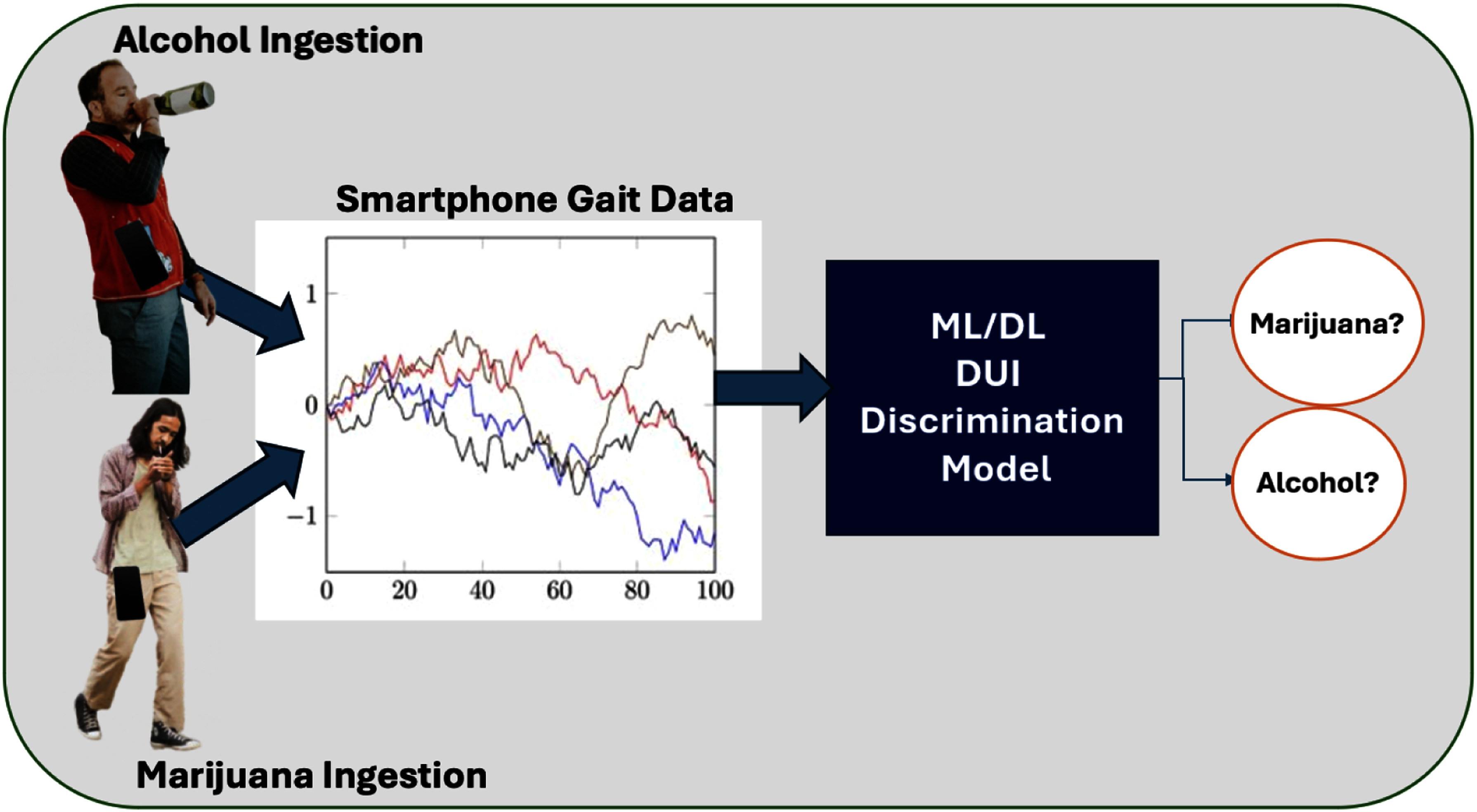
High-level overview of our approach for alcohol-marijuana discrimination.

*Our approach:* This paper focuses on discriminating two classes: alcohol and marijuana-induced gait impairments. Fig. 1 illustrates *MariaGait*, our machine learning framework to detect alcohol or marijuana impairment from smartphone sensor-based gait data (accelerometer and gyroscope). In real-world deployments, this gait data can be passively collected and analyzed. If impairment is detected, interventions such as subject DUI alerts, vehicle immobilization, or ride-hailing can be triggered. Our method utilizes a tiled CNN with TICA pooling for classification. Inspired by prior research [27], [33], [34], [35], [36], [37], we first encode the time-series gait data as Gramian Angular Field (GAF) images. The tiled CNN divides these images into tiles, sharing weights across features to reduce parameters and enhance invariance learning. Our tiled convolution to learn localized, orthogonal features from encoded gait signals and TICA pooling technique is conceptually analogous to adaptive diffusion priors and physics-driven reconstruction techniques used in small-data medical imaging approaches such as Parallel-stream fusion [39], Adaptive Diffusion Priors [40], Autoregressive State Space Models [41]. These ideas influenced our model pre-training on sober samples to increase model generalization and performance.

*Prior work:* Similar methods have been successful in time-series classification tasks such as Human Activity Recognition (HAR) [33], [34], frailty assessment [36], and gait-related disease diagnosis [38] but not alcohol vs. marijuana DUI discrimination. Prior studies have demonstrated that neural networks analyzing GAF images [42] outperform those without GAF [28], [37], [43], [44], as well as traditional models [45], [51], [52], [53].

For gait-specific tasks, an MS-CNN analyzing GAF-encoded images achieved superior accuracy and precision in frailty detection, outperforming STFT and CWT time-series encodings [27], [36]. However, since GAF images lack natural image elements such as edges and angles, simple CNNs struggle with feature extraction. To address this, Zhiguang proposed TICA pooling layers [54] and a stacked tiled CNN model for image matching. Our approach integrates a single-tiled CNN with TICA pooling, an unsupervised algorithm that pools related features while maintaining orthogonality. This enhances feature diversity, reduces redundancy, and improves model robustness. Additionally, TICA pooling enables pre-training on more abundant sober gait samples to improve classification of alcohol- and marijuana-induced impairment and solve the problem class imbalance caused with limited impaired samples.

*Prior work on gait analysis to detect impairment from consuming alcohol or marijuana:* Table I summarizes prior methods for detecting alcohol and marijuana impairment from various different data types and machine learning models. Prior gait analyses work focused on three types of data: 1) videos from cameras, 2) pressure-sensors embedded in shoes and 3) IMU times series sensor signals from ubiquitous devices such as smartphones and wearables [35], [55], [56].

**TABLE I:** Prior Work on Impairment Classification

					
(a) Prior work on alcohol impairment classification from gait					
**Method type**	**Method**	**Features**	**Accuracy**	**Target classes**	**Source**
**Traditional ML**	RF	(SNR, Cadence, Skewness, Kurtosis)	70%	Sober(0.2 drinks), tipsy(3-6 drinks), drunk (above 6 drinks)	Arnold et al. [45]
	J48 classifier	(Sway Area, Sway Volume, Kurt, Skew, Gait Velocity)	89.45%	[0.00-0.08), [0.08-0.15), [0.15-0.25), [0.25+)	Aiello & Agu [46]
**Deep Learning**	Bi-CNN	Raw sensor data	83.5%	Sober, drunk	Li et al. [27]
(b) Prior work on marijuana impairment classification from various modalities					
**Method type**	**Method**	**Data Type**	**Accuracy**	**F1 score**	**Source**
**Deep Learning**	2-layer 2D CNN	Speech	——	Females: 68.6%, Males: 67.9%	Sobieraj et al. [47]
	CNN	Facial Images	73%	0.71	Gadhiya et al. [48]
	VGG-16	Facial Images	94.66%	0.9292	Jain et al. [23]
	LGBM	Time features	60%	0.64	Bae et al. [49]
		Smartphone sensor data + time features	90%	0.90	Bae et al. [49]
	RNN + XGBoost	fNIRS + time course features	76.4%	————	Gilman et al. [50]

**Our key contributions** are as follows:

• We propose *MariaGait*, a Topographic Independent Component Analysis (TICA) multi-head Tile CNN architecture for impairment substance classification of a GAF image representation of smartphone sensor gait data. *MariaGait* is shown in Fig. 3 A TICA pooling CNN layer that is pre-trained on more abundant sober gait data is utilized for overcoming an imbalanced dataset, improving performance. Prior work did not leverage orthogonal pooling techniques such as TICA to improve feature separation and reduce overfitting caused by a small dataset.
• To the best of our knowledge, our study is the first work to explore GAF image-based encodings to transform time-series sensor data into an image for alcohol vs. marijuana substance intoxication discrimination.
• We performed rigorous evaluation of the proposed *MariaGait* method, which achieved 94.61% accuracy, F1 score of 88.61%, and 94.33% ROC AUC score for the binary classification of whether the subject consumed alcohol or marijuana. The proposed method outperformed a comprehensive set of state-of-the-art (SOTA) baseline models for this domain including neural network models (Multi-Layer Perceptron (MLP), CNN, CNN with GAF, Long Short Term Memory (LSTM), Multi-head LSTM) as well as traditional machine learning models (Random Forest and Support Vector Machines(SVM)) [57]. Multi-head CNN and multi-head LSTM models are also included as baselines. The multi-head CNN/multi-head LSTM [58] is another popular neural network architecture that is used for time-series classification. Recent SOTA architectures such as adaptive diffusion priors, attention-guided CNNs, and transformer-based models have been explored in medical signal reconstruction and low-data learning scenarios [40], [59], [60]. Although these methods were developed for different domains, their architectural principles inspired our use of TICA pooling and tiled CNN design. Notably, while attention-guided CNNs were originally proposed for natural images, their core mechanism—spatially attending to salient regions—are broadly applicable to time-series encodings such as GAF, where temporal relationships are preserved in a spatial representation.

The rest of this paper is as follows. Technical details, background information and the disadvantages and advantages of this proposed model will be introduced in Section II.

## II. Materials and Methods

### A. Data Collection for Alcohol and Marijuana-Impaired Gait

Gait data from 111 participants was collected in two NIH-funded studies on alcohol and marijuana impairment. 101 subjects completed a walking task after alcohol consumption, while 10 subjects participated after marijuana use. Both studies followed the same protocol, where participants walked 75 to 150 feet multiple times while carrying a smartphone that recorded accelerometer and gyroscope data.

Alcohol doses were adjusted based on breathalyzer readings, while marijuana participants smoked cigarettes with 0%, 3%, or 7.2% THC, completing walking tasks at multiple intervals post-consumption. Further details are provided in the Supplementary Materials.

### B. Data Preprocessing

**Fig. 2.**
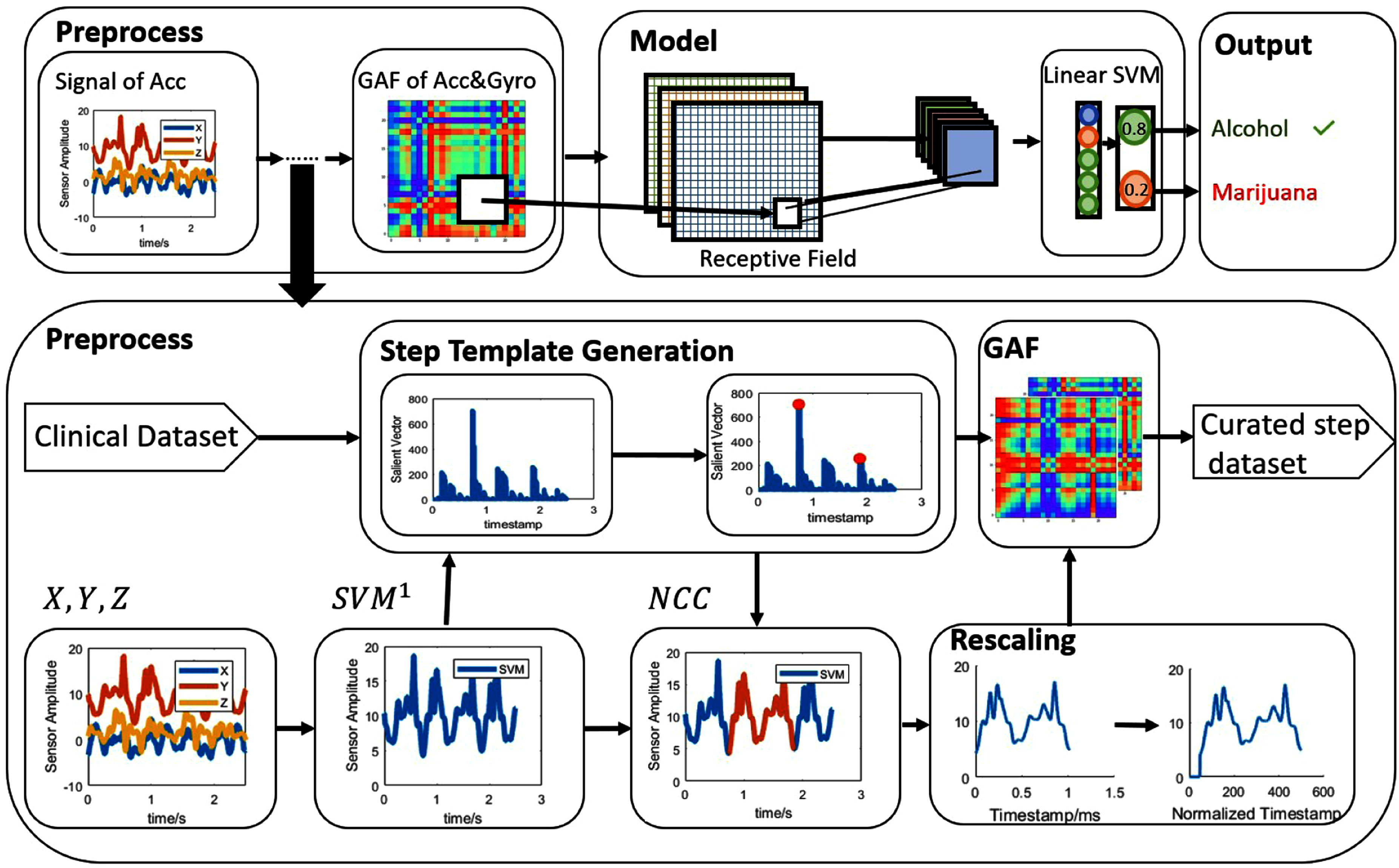
Data flow in our *MariaGait* methodology, illustrating the main steps involved including collecting triaxial smartphone accelerometer and gyroscope sensor data, sensor signal preprocessing, and classification using a Tile CNN with TICA pooling predictive model. The blocks below illustrate the techniques utilized for the signal processing step. The input dataset includes 1071 accelerometer time series signals, which are described in Table S1 in the Supplementary Materials. $SVM^{1}$ denotes the signal vector magnitude, which is calculated as the square root of the sum of the three axes. Utilizing $SVM^{1}$ enabled segmentation of the time series into cycles using sequential salient points. Salient points are data samples with local minimum amplitude [61]. NCC denotes Normalized Cross-Correlation, which represents the similarity between the template gait and the target segment. High values in the correlation map indicate a strong similarity between the template and the target region, suggesting the target segment is a subject's other gait cycle. Algorithm 1 in the Supplementary Materials shows our gait signal preprocessing algorithm. The gait time-series samples are transformed into GAF images. The output dataset includes 21070 Gramian Angular Fields (GAF) images of dimensions 500 pixel x 500 pixel x 3 axes. The arrows represent the flow of data and decisions. For additional description of the classification model, Fig. S6 presents the structure of the tiled layer, and Fig. S5 and S7 in the Supplementary Materials, present two types of tiled CNNs. Table S2 in the Supplementary Materials shows parameters of the layers.

Preprocessing of raw accelerometer and gyroscope data involved filtering, outlier removal, signal segmentation, and GAF encoding for classification using the multi-head tile TICA CNN model (Fig. 2). Our data preprocessing pipeline is extensively discussed in the Supplementary Materials.

**Fig. 3.**
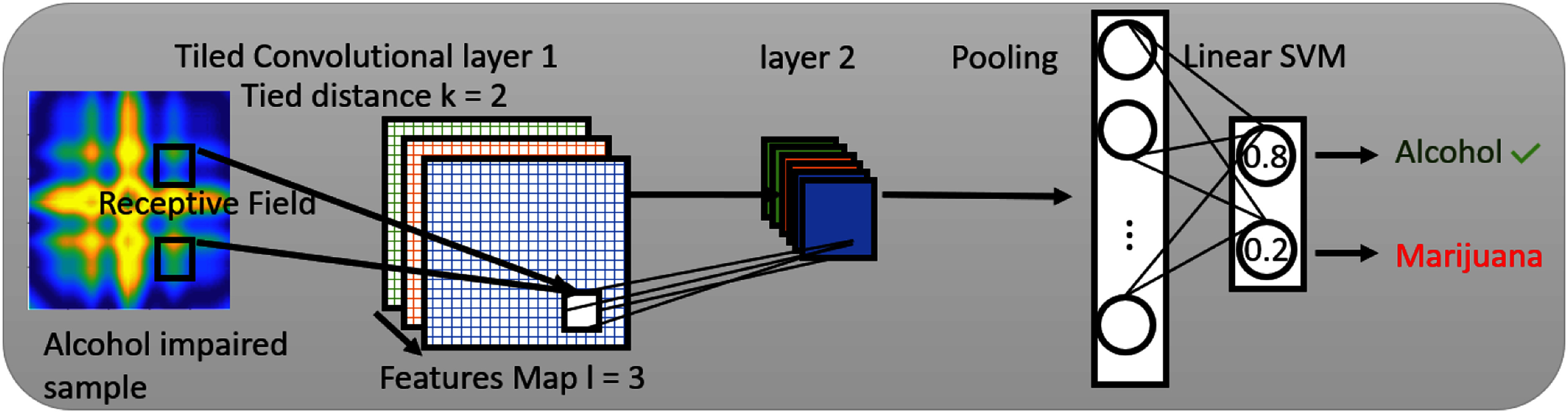
Pre-trained TICA Tile CNN Model.

**Fig. 4.**
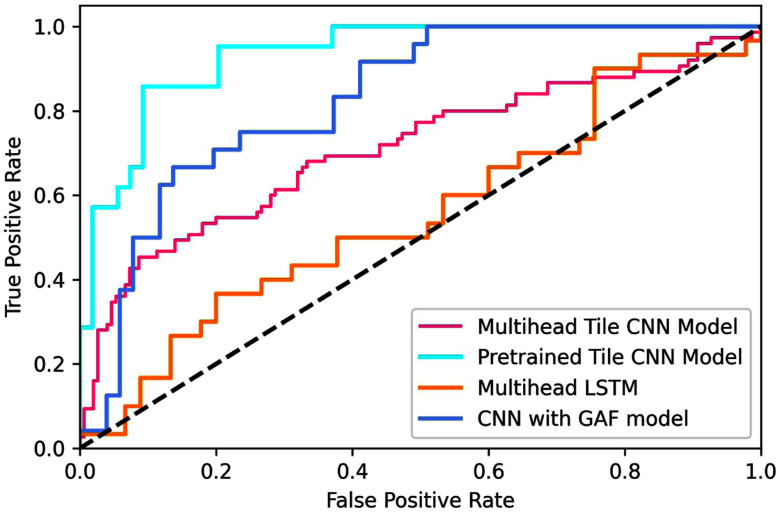
ROC curves of our proposed *MariaGait*. approach and 3 state-of-the-art baseline models.

### C. Tile CNN Layer

Initially proposed by Quoc V. Le [63], the tile CNN significantly outperformed a regular CNN and Support Vector Machine (SVM) for image classification tasks on both the NORB and CIFAR-10 datasets [64], which has made it popular recently [65]. Moreover, the tiled CNN has been more broadly applied to sensor signal processing [54] and natural language processing [66]. The tile layer is a strategy that ties the weights (“tiling”) of several features in a feature map. Utilizing tiling techniques benefited our proposed model in two main ways [64]. First, tiling reduces the number of learnable parameters in each feature map since traditional CNNs learn basis functions at various locations and ties them all together. However, in tiled CNN, units with hard-coded distance $k$ and multiple $k$ steps share tied weights. Then, two units at the distance $k$ are untied. As presented in Fig. S6 in the Supplementary Materials, units with the same fill texture share tied weights. Secondly, efficient tiling makes it possible for the learning algorithm to learn more invariances from different maps, since different maps will yield different sets of learned parameters. In our experiments, each convolutional tiled layer was set up with 3 feature maps and a tile distance of 2 as shown in Fig. S6 in the Supplementary Materials. In addition to this standard configuration, we evaluated a pre-trained tiled CNN and a Multi-head tiled CNN. The pre-trained tiled CNN includes a TICA layer, consisting of a 3-layer, 2-distance tiled convolutional layer followed by TICA pooling. The Multi-head tiled CNN is a more complex architecture that combines three tiled CNNs, followed by a concatenated TICA pooling layer, without any pre-training. Both models were evaluated against baseline models, including LSTM, Multi-LSTM, CNN, CNN with GAF, Random Forest (RF), and SVM. The performance of these models was evaluated using the F1 score and accuracy metrics, and ROC curves were generated to further illustrate their effectiveness.

### D. Tile CNN With Tica Pooling Neural Networks Model

#### 1) TICA Pooling

Algorithm 1: **Unsupervised Pretraining of Tiled CNNs With TICA (Line Search).**
**Require:** $\lbrace x^{(t)}\rbrace _{t=1}^{T}, W, V, k, s$ $\triangleright$$k$ is the tile size, $s$ is the receptive field size
**Ensure:** $W$
1: **repeat**
2:
$f^{\text{old}} \gets \sum _{t=1}^{T} \sum _{i=1}^{m} \sqrt{\sum _{k=1}^{m} V_{ik} (\sum _{j=1}^{n} W_{kj} x_{j}^{(t)})^{2}}$

3:
$g \gets \frac{\partial [\sum _{t=1}^{T} \sum _{i=1}^{m} \sqrt{\sum _{k=1}^{m} V_{ik} (\sum _{j=1}^{n} W_{kj} x_{j}^{(t)})^{2}}]}{\partial W}$

4:
$f^{\text{new}} \gets +\infty, \alpha \gets 1$

5: **while** $f^{\text{new}} \geq f^{\text{old}}$ **do**
6:
$W^{\text{new}} \gets W - \alpha g$

7:
$W^{\text{new}} \gets \mathtt {localize}(W^{\text{new}}, s)$

8:
$W^{\text{new}} \gets \mathtt {tie\_{w}eights}(W^{\text{new}}, k)$

9:
$W^{\text{new}} \gets \mathtt {orthogonalize\_{l}ocal\_{R}F}(W^{\text{new}})$

10:
$f^{\text{new}} \gets \sum _{t=1}^{T} \sum _{i=1}^{m} \sqrt{\sum _{k=1}^{m} V_{ik} (\sum _{j=1}^{n} W_{kj}^{\text{new}} x_{j}^{(t)})^{2}}$

11:
$\alpha \gets 0.5\alpha$

12: **end while**
13:
$W \gets W^{\text{new}}$

14: **until** convergence

Similar to other pooling strategies, TICA pooling is utilized for pooling convolutional layer outputs as well. The equation for Orthogonal TICA pre-training is presented in (1).

\begin{equation*}
W_{minimize} \sum _{t=1}^{T} \sum _{m=1}^{M} p_{i} (x^{t};W,V),WW^{T}=1 \tag{1}
\end{equation*}

In (1) above, $m$ denotes the feature map, $W$ denotes the weight and $V$ denotes the vector of hidden values. Like other feature maps in deep learning, the learned TICA weight vector maps try to pool together groups of related features, while maintaining orthogonal relationships in features maps, $W*W^{T}=1$. Thus, those feature vector maps are perpendicular to each other and highly independent. Highly independent weight vector maps reduce redundancy, enhance diverse feature extraction, and improve model robustness, yielding more efficient and accurate representations. As expressed in (1), TICA pooling is an unsupervised learning algorithm that is able to learn features from unlabeled image patches [64]. In most clinical and bio-informatics research, researchers have to address the lack of positively labeled (impaired) samples. Classifying ingested substances from gait is no exception. To avoid collecting more labeled data from the real world, many researchers have utilized approaches such as data augmentation and pre-training. In our case, utilizing TICA pooling made it possible to pre-train the neural networks model on sober gait samples that were easier to collect and hence more abundant to improve performance on our primary task of classifying ingested substance (alcohol or marijuana). The original algorithm used by Ngiam, J et al. [64] for pretraining is shown in algorithm 1, which utilizes gradient descent on the TICA objective (1).

\begin{equation*}
p_{i}(x^{(t)}; W, V) = \sqrt{\sum \nolimits_{k=1}^{m} V_{ik} \left(\sum \nolimits_{j=1}^{n} W_{kj} x_{j}^{(t)} \right)^{2}}. \tag{2}
\end{equation*}

where (2) shows the activation of each second-layer unit for an input pattern $x^{(t)}$

## III. Results

### A. Evaluation Metrics

We evaluated model performance using F1-score and ROC-AUC score for imbalanced data, along with accuracy. Key baselines are compared across accuracy, and where available, F1 and AUC scores. All reported metrics refer to the model's performance in discriminating between the two classes: alcohol and marijuana. Detailed explanation and equations are presented in Supplementary Materials.

### B. Experiments

*Train-test curves to confirm no overfitting:* Comparing the accuracy of the train vs. test curves shown in Fig. S8 in the Supplementary Materials reveals a small gap between training and test accuracies, demonstrating that our proposed *MariaGait* tile CNN with Tica pooling model did not overfit. Table S2 and Fig. S5, which shows the CNN model constructure of multihead tile, are shown in the Supplementary Materials.

*Comparison of the MariaGait* Tile CNN with Tica pooling model with baselines: Five substance impairment prediction models were developed as baselines including MLP, LSTM, Multi-head CNN and Multi-head LSTM as well as traditional machine learning models (Random Forest and SVM). More details on the baseline models are presented in the Supplementary Materials. These baselines previously achieved good results on smartphone sensitivity of biphasic user impairment from different kinds of walks [57]. Three advanced models were also developed and compared including the Multihead LSTM, pre-trained TICA Tile CNN with GAF and Multihead Orthogonal Tiled CNN with GAF.

Based on the results summarized in Table II, the pre-trained TICA tile CNN with GAF (*MariaGait*) model outperformed other models, achieving an accuracy and F1-score of 94.61% accuracy and 88.61% respectively. As shown in Fig. 4, *MariaGait* outperformed the advanced baselines with an impressive AUC-ROC score of 94.33% showing its strong ability to discriminate alcohol impairment from marijuana impairment. Overall, models that analyze GAF images (CNN with GAF, TICA CNN with GAF and Multihead CNN with GAF) significantly outperformed models that did not utilize GAF (LSTM, MLP, Random forest and Multihead LSTM). Multihead models (Multihead LSTM and Multihead CNN), which are more complex and incorporate more variables to be learned, did not have conclusively better performance. The pre-trained (*MariaGait*) tile CNN outperformed the multi-head tile CNN. Besides, compared to other traditional methods, the multi-head LSTM did not achieve the accuracy achieved by baseline models (LSTM and MLP).

**TABLE II:** Results of Marijuana and Alcohol Impairment. (*multihead LSTM is Used for Accelerometer Time-Series Data Gait Analysis and Achieved the Best Performance; *orthogonal Tiled CNN is an Advanced Model for the Image Classification Task. *CNN+GAF Achieves the Best Performance in the Alcohol Substance Impairment Detection Task.)

Experiments Performance Metrics			
Model	Accuracy	F1-Score	ROC AUC
Long Short Term Memory(LSTM)	72.56%		
Multi-Layer Perceptron(MLP)	90.34%		
*CNN with GAF	**91.25**%		
CNN	83.67%		
Random Forest	82.91%		
Multihead LSTM [58]	68.30%	67.57%	71.30%
**Pretrained TICA(Orthogonal) Tile CNN+GAF** *(MariaGait)*	**94.61%**	**88.61%**	**94.33%**
Multi-head Tiled CNN+GAF [54]	92.33%	86.3%	92.02%

*Examining the utility of multi-head model structure:* Table II compares various variants of the proposed approach using three metrics for the impaired substance prediction task. Unlike the original muti-head LSTM research [58], the multi-head LSTM does not achieve a convincing performance in our DUI discrimination task. We believe that this is because in the original multi-head LSTM research, five types of sensors were utilized, instead of triaxial data from three axes of a single sensor. The feature vectors input to the three heads input had a strong linear combination property, which caused redundancy in learnable parameters and reduced the ability of subsequent RNN layers to learn. Inspired by multi-head LSTM models proposed by others, in future work, gyroscope sensors could be introduced on the $2^{nd}$ head of LSTM in addition to the $1^{st}$ accelerometer head of LSTM.

## IV. Discussion

### A. Main Findings

*The proposed MariaGait* tile CNN with Tica pooling model achieved clinically usable performance (94.61% accuracy, 0.8861 f1 score and 0.94 AUC score) which was encouraging. This demonstrates the feasibility of utilizing deep learning methods for prediction of various impairment substances such as recreational drugs, prescription medications and classification of symptoms of user impairment [67]. Based on the exposition in Section III-B, we can make the following conclusions:

1) *The tile CNN architecture with TICA pooling mechanism improved DUI discrimination accuracy* outperforming a CNN with GAF and no tile CNN and TICA pooling by 3.36% in accuracy. This demonstrates the validity of two of our key ideas. Tiling improves model performance by forcing the learning of invariant features across tiles. By focusing on local patterns within tiles, TICA pooling improves model performance because it learns spatiotemporal features from sequential datasets such as time series datasets compared to other pooling mechanisms such as maxpooling and average pooling.
2) *Encoding sensor data as a GAF image generally improves the accuracy of the substance classification models* because compared with analyzing the original time-series dataset, the encoding imaging dataset provides more learnable variables and achieves higher accuracy; Specifically, CNN+GAF, Multi-head Tile CNN+GAF and Pretrained TICA (Orthogonal) Tile (*MariaGait*) CNN+GAF achieved 91.25%, 92.33% and 94.61% accuracies respectively.
3) *Multi-head models did not conclusively achieve higher accuracy:* even though multi-head models provide more learnable variables for both time-series and image datasets. Specifically, multi-head LSTM and Multi-head Tiled CNN+GAF achieved 68.30% and 92.33% accuracies respectively, which were 26.31% and 2.28% lower than the 94.61% accuracy achieved by the Pretrained TICA (Orthogonal) Tile (*MariaGait*) CNN+GAF.
4) *Pre-training the CNN architecture on sober gait samples further improved model performance* In fact, pre-training boosted performance more than the multi-head approaches as the pre-trained tiled CNN model performed better than the multi-head model based on both the accuracy and ROC metrics. The Pre-trained Tile CNN with TICA pooling (*MariaGait*) model achieved an accuracy of 94.61% and an AUC score of 0.94, outperforming the next best performing model by $>2.28\%$ accuracy and $>2.31\%$ AUC score.

### B. Study Limitations

#### 1) Insufficient Data

The dataset comprises 13,211 impaired gait samples, which may be insufficient for training complex models like the tiled CNN. While traditional models can perform well with smaller datasets, deep learning models benefit from larger, more diverse data for improved feature learning and generalization. Although pre-training on a larger sober dataset mitigated this limitation, a more extensive and balanced dataset would enhance performance. Future work could address this using synthetic data generation (SMOTE, GANs) and augmentation (Rotation, Scaling).

Another limitation is the inability to distinguish impairment in users who consume both alcohol and marijuana simultaneously. Given the prevalence of polysubstance use and its potential to intensify impairment, future research will focus on addressing this issue.

#### 2) Future Work

Additional analysis is necessary for future research in order to confirm our findings, particularly more machine learning studies on new datasets. Alternate approaches also need to be investigated for the components of our proposed framework including: a) Our step segmentation method needs to be compared to other segmentation methods. b) Normalization of other subject attributes such as age, height, weight, and Body Mass Index (BMI), need to be explored, and c) The GAF imaging approach needs to be compared to other time-series imaging methods such as Markov Transition Fields (MTF) and Recurrence Plots (RP), and d) the performance of our models in live deployment needs to be evaluated. e) lightweight transformer-based CNN hybrids and attention-guided pooling mechanisms or hybrid attention-CNN architectures with contrastive self-supervised learning would be explored as part of our future directions to further improve efficiency, generalization and accuracy in low-data regimes.

### C. Computational Complexity Analysis

of MariaGait is presented extensively in the Supplementary Materials document.

## V. Conclusion

In this work, using first-of-a-kind impaired gait datasets, we proposed *MariaGait*, a novel approach to distinguish alcohol- and marijuana-induced impairment from smartphone inertial sensor data. *MariaGait* innovatively combines step-segmented gait signals, Gramian Angular Field (GAF) encoding, and a Tiled CNN with TICA pooling and model pre-training on sober gait samples. This work is the first to discriminate between alcohol and marijuana impairment using deep learning on gait-based smartphone data. It achieved 94.61% classification accuracy, 88.61% F1 score and 94.33% AUC ROC score, significantly outperforming traditional machine learning classifiers (SVM, Random Forest) and modern deep architectures (LSTM, multi-head CNN). These results highlight the value of combining visual time-series encoding with advanced CNN architectures and pooling strategies. These findings demonstrate the model's potential for real-world, non-invasive DUI detection and substance abuse monitoring, and its adaptability to broader applications such as movement disorder assessment. To the best of our knowledge, this is the first study to distinguish alcohol- and marijuana-induced gait impairments using a pre-trained, orthogonalized Tile CNN on GAF-encoded smartphone sensor data — a novel approach suited to data-constrained problems.

## Supplementary Materials

Supplementary details, including extended methodologies, additional figures, and key equations, to support the findings presented in the main text are available in the Supplementary Materials document. Supplementary materials are provided as a separate document.

Supplementary Materials

## Conflicts of Interest

The authors declare that they have no conflict of interest.

## Author Contributions

R. Li contributed to the conception, design, manuscript drafting, code writing and data analysis of the study. S. C. Uche contributed to manuscript drafting, review and editing, literature review, data and results visualization. E. Agu contributed to supervision, resources, funding acquisition and project administration. K. Grimone, D. S. Herman, J. Metrik, A. M. Abrantes, and M. D. Stein contributed to formal data collection. All authors read and approved the submitted manuscript.
