# Supplementary material for: Discriminating Between Marijuana and Alcohol Gait Impairments Using Tile CNN With TICA Pooling
Source: IEEE Open J Eng Med Biol. 2025 Sep 9;6:540–8. doi: 10.1109/OJEMB.2025.3607556 (PMC12599891; doi:10.1109/OJEMB.2025.3607556)
Supplement: Supplementary Materials [file supp1-3607556.pdf]

Supplementary Materials

Discriminating between Marijuana and Alcohol Gait Impairments using Tile CNN with TICA Pooling

Ruojun Li, *student Member, IEEE,* Samuel Chibuoyim Uche, *student Member, IEEE,* Emmanuel Agu, *Member, IEEE*, Kristin Grimone, Debra S. Herman, Jane Metrik, Ana M. Abrantes and Michael D. Stein

I. MATERIALS AND METHODS

A. Alcohol and Marijuana Impaired Gait Data Collection:

Our alcohol and marijuana gait datasets were collected by a human proctor from a total of 111 subjects who participated in two NIH-funded studies. 101 of these subjects completed the gait task in which they consumed and were impaired by alcohol and 10 subjects participated in gait tasks in which they were administered and were impaired by marijuana. Both experiments utilized the same walking protocol and were conducted on the premises of Butler Hospital and Brown University for the alcohol and Marijuana data collection studies respectively, with distances of 75-feet (one direction) and 150-feet (back and forth) for each walk. Each subject repeated the walking protocol 10-30 times in the alcohol protocol and a few supervised walks in the marijuana protocol. During each walk, subjects took 50 to 100 seconds to walk with a smartphone in their pocket. An Android sensor data gathering application was programmed to collect accelerometer and gyroscope sensor data from a smartphone carried by the subject. Specifically, linear acceleration along one or multiple axes, static acceleration, gravity, and dynamic acceleration which results from the object's motion gait, were all collected. More details on the substance administration protocols are provided in the following sections.

1) Alcohol Protocol: The alcohol data gathering study was funded by the National Institute of Health (NIH) and the National Institute on Alcohol Abuse and Alcoholism (NIAAA) under Grant 1R21AA025193-01. All experimental procedures and protocols adhered to ethical standards and were approved by the Institutional Review Board (IRB) at Butler Hospital in Providence, Rhode Island [1]. All participants received Hurricane Beer, having 8.1% alcohol content. Each participant started with either 3 or 4 ounces of alcohol, depending on weight (men over 150 lbs and women over 160 lbs received 4 ounces initially). A BAC measurement was taken 15 minutes later using a breathalyzer. The amount of alcohol each participant received subsequently depended on each BAC reading. If an individual ascended at an even pace (e.g., .02 BAC with each drink), they continued to receive the same amount of alcohol. If an individual's BAC rose more than approximately .02, or if the BAC came very close but did not fully reach 0.10, less alcohol was provided at the next alcohol administration. Increasing the amount of alcohol administered per drink was also possible but rarely occurred.

2) Marijuana Protocol: As part of the larger parent study at Brown University (RO1 AA024091, PI Metrik), participants (N = 10) completed three double-blind experimental laboratory sessions following 15-hour abstinence from marijuana, in which they smoked a marijuana cigarette with 3% THC, 7.2% THC, and placebo (0% THC), with order of administration counter-balanced across subjects. Participants completed a brief walking task before they smoked and then approximately 15, 40, and 60 minutes after they smoked the assigned dose, while a smartphone app gathered their gait data.

B. Data Preprocessing

Preprocessing steps for raw accelerometer and gyroscope data included filtering, outlier removal, signal segmentation, GAF encoding of the smartphone sensor time series data into an image for eventual classification using the multi-head tile TICA CNN model, which are illustrated in Fig. 2 in the main paper. Algorithm 1 summarizes our preprocessing pipeline also captured in Fig. 2 in the main paper.

The Signal Vector Magnitude (SVM) of the triaxial accelerometer and gyroscope time series sensor data was computed, giving a robust pipeline that is invariant to device orientation. The computed SVM was utilized in the template generation step. In this step, templates representing repeating patterns were created for a single gait cycle based on the SVM signal. Each template is a segment of the signal defined by two consecutive minimum salient points, with each template approximately corresponding to one gait cycle. Salient points are data samples with local minimal amplitudes, and the method for identifying these points is described in detail in [2]. Next, the Normalized Correlation Coefficient (NCC) was computed. In this step, the normalized correlation of triaxial accelerometer data was used to detect the peaks and troughs of the SVM of the accelerometer and gyroscope signal. Equations (1), (2), and (3) show the mathematical formulas for SVM, cross-correlation coefficient and normalized correlation coefficient of the accelerometer and gyroscope signal.

In Equation (2), $B_r(k)$ represents the *reference gait segment*, while $B_s(k+i)$ denotes a *shifted target segment* of the signal. These segments are extracted using the salient points and are used to compute the normalized cross-correlation (NCC) for gait cycle matching. It is instructive to note that while gait cycle analysis has traditionally analyzed data gathered from foot-mounted sensors, recent literature confirms that smartphone-based gait analyses using data from smartphones placed in pockets or on thighs captures reliable periodic gait signatures suitable for segmentation and analyses [3]. In our study, segmentation is based on local minima in the Signal Vector Magnitude (SVM), yielding segments with consistent and repeatable motion even if not aligned to formal heel-strike

events. We utilize these segments for GAF transformation and classification.

Algorithm 1 Gait Signal Preprocessing Algorithm

procedure COMPUTE SVM(acc_x, acc_y, acc_z)
  $SVM_{acc} \leftarrow \sqrt{acc_x^2 + acc_y^2 + acc_z^2}$
  return SVM_{acc}
end procedure
procedure IDENTIFY SALIENT POINTS(SVM)
  Find local minima $\{p_i\}$ where $SVM(p_i) < SVM(p_{i-1})$ and $SVM(p_i) < SVM(p_{i+1})$
  return salient points $\{p_i\}$
end procedure
procedure TEMPLATE GENERATION(SVM, salient points $\{p_i\}$)
  for $i = 1$ to $N-1$ **do**
    Extract segment T_i between p_i and p_{i+1}
  end for
  return templates $\{T_i\}$
end procedure
procedure COMPUTE NCC(B_r, B_s)
  for $i = 0$ to $L - M$ **do**
    $D(i) \leftarrow \frac{\sum_{k=1}^{M} B_r(k) B_s(k+i)}{\sum_{k=1}^{M} B_r(k)^2}$
  end for
  return cross-correlation coefficients $D(i)$
end procedure
procedure NORMALIZATION($signal$, Target Length)
  $siglen \leftarrow$ length of $signal$
  if $siglen >$ Target Length **then**
    $r \leftarrow$ Target Length / $siglen$
    return $interp(signal, r)$
  else
    return $signal(0:$ Target Length$)$
  end if
end procedure
procedure GAF TRANSFORMATION(X)
  for i, j in $[1, N]$ **do**
    $\Phi_i = \arccos(X_i)$
    $GAF_{i,j} = \sin(\Phi_i + \Phi_j)$
  end for
  return GAF
end procedure

$$SVM_{acc} = \sqrt{acc_x^2 + acc_y^2 + acc_z^2} \tag{1}$$

where x, y, z are the triaxial features of the accelerometer time series data.

$$D(i) = \frac{\sum_{k=1}^{M} B_r(k) \cdot B_s(k+i)}{\sum_{k=1}^{M} B_r(k)^2}, \quad i \in [0, L-M] \tag{2}$$

- $D(i)$: Cross-correlation coefficient at lag i, measuring the similarity between B_r and B_s with B_s shifted by i.
- $B_r(k)$: Reference signal value at position k.
- $B_s(k+i)$: Shifted signal value at position $k+i$.
- **Numerator**:
$$\sum_{k=1}^{M} B_r(k) \cdot B_s(k+i)$$
This is the dot product between the reference and shifted signal.
- **Denominator**:
$$\sum_{k=1}^{M} B_r(k)^2$$
This is the normalization term for the reference signal.
- **Range of** i: $i \in [0, L-M]$, where L is the length of B_s and M is the length of B_r.

$$NCC(i) = \frac{\sum_{k=1}^{M} \left(B_r(k) - \bar{B}_r\right)\left(B_s(k+i) - \bar{B}_s(i)\right)}{\sqrt{\sum_{k=1}^{M} \left(B_r(k) - \bar{B}_r\right)^2 \sum_{k=1}^{M} \left(B_s(k+i) - \bar{B}_s(i)\right)^2}}$$
$$\text{where } i \in [0, L-M] \tag{3}$$

- $NCC(i)$: Normalized cross-correlation coefficient at lag i, measuring the similarity between the reference signal and a shifted segment of the target signal.
- $B_r(k)$: Reference signal value at index k.
- $B_s(k+i)$: Signal value from the target sequence, shifted by i.
- $\bar{B}_r$: Mean of the reference signal segment.
- $\bar{B}_s(i)$: Mean of the segment of the target signal B_s starting at lag i.
- **Numerator**: Computes the dot product between the mean-centered reference and shifted target segment.
- **Denominator**: Normalizes the result by the product of their standard deviations.
- **Range of** i: $i \in [0, L-M]$, where L is the length of the target signal and M is the length of the reference template.

Algorithm 2 shows our unique time normalization algorithm. This algorithm standardizes the gait data into a uniform length and scale, as deep learning models require preprocessed input data to be of uniform length. This is the last step before the data is encoded into GAF images.

1) Gramian Angular Field (GAF) Transformation: GAF transformation is an algorithm for encoding times series signal into polar coordinates, transforming the sensor data into an image, as presented in Fig. S1. [4] The main GAF equations are given in equations (4) and (5).

$$\begin{cases} \Phi_i = arccos(x_i) \\ r_i = \frac{i}{N} \end{cases} \tag{4}$$

$$GAF_{i,j} = sin\left(\Phi_i + \Phi_j\right) \tag{5}$$

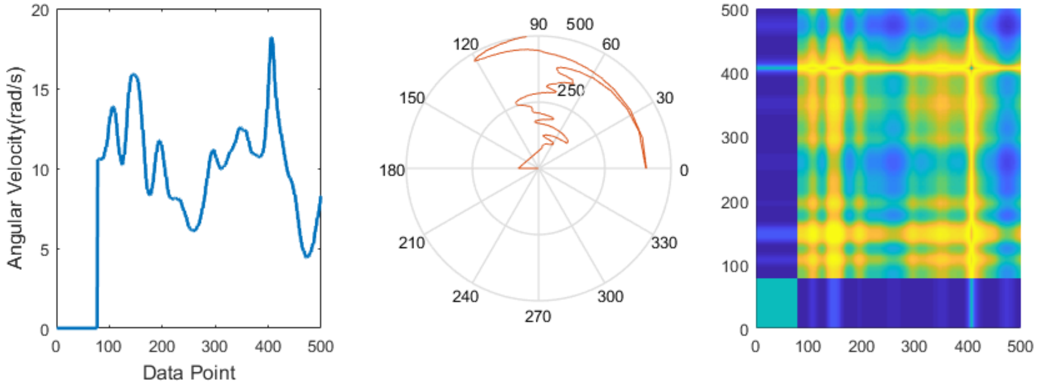

Fig. S1. Illustration of transformation of a smartphone sensor signal into a GAF-encoded image

Algorithm 2 Normalization

procedure INTERPOLATION
    $signal \leftarrow$ sensor signal after mid-swing
    $siglen \leftarrow$ length of $signal$
    $len \leftarrow$ Target Length - First Swing Phase Length
    output: signal after first swing
    if $siglen > len$ **then**
        $r \leftarrow len/siglen$
        return $interp(signal, r)$
    else
        return $signal(0 : len)$
    end if
end procedure
procedure FIRST SWING PHASE
    $signal \leftarrow$ sensor signal of First swing
    $siglen \leftarrow$ length of $signal$
    $len \leftarrow$ Target First Swing Phase Length
    output: Signal of first swing
    if $siglen < len$ **then**
        return $zeros(len - siglen) + signal$
    else
        return $signal(siglen - len : siglen)$
    end if
end procedure
procedure MAIN NORMALIZATION
    $signal \leftarrow$ Signal of first swing + signal after first swing
    $ratio \leftarrow \frac{(Attribute - Attribute_{min})}{(Attribute_{max} - Attribute_{min})}$
    output: Normalized Signal
    if length of $signal$ = Target Length **then**
        return $signal * ratio$
    else
        return IllegalSampleError
    end if
end procedure

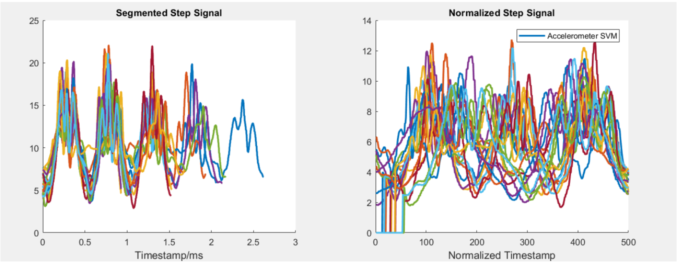

Fig S2: Samples of data with subjects impaired by Marijuana. The x-axis Timestamp units is in milliseconds

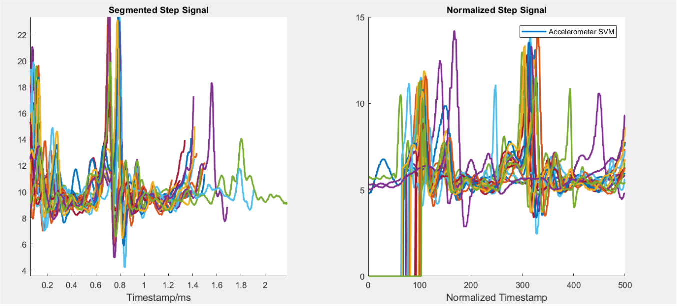

Fig S3: Samples of data with subjects impaired by Alcohol. The x-axis Timestamp units is in milliseconds

Utilizing the segmentation process outlined in Fig. 1 in the main paper, 13211 samples were extracted from 1071 walks of 111 subjects. Samples of subjects impaired by consuming either marijuana or alcohol are presented in Fig. S2 and Fig. S3. Table S1 shows the distribution of the dataset. Fig. S4 shows the distribution plot of our data set. In order to pre-train the TICA layer, sober gait samples were also included in order to create a negative class as well for machine learning classification. To preprocess the gait data, the same workflow shown in Fig. 1 in the main paper was utilized. The preprocessing method was able to improve discrimination between marijuana and alcohol significantly, as illustrated in Fig. S2 and Fig. S3. Our study noted inter-subject variability in signal shape which reflects an expected variation in gait mechanics and impairment effects. This diversity is handled by normalization and GAF encoding prior to classification.

TABLE S1. Summary of valid Alcohol and Marijuana Gait Datasets

Substance	Subjects	Walks	Samples
Alcohol Impaired	101	749	9180
Marijuana Impaired	10	151	4031
Pretrained Sober	131	171	7859

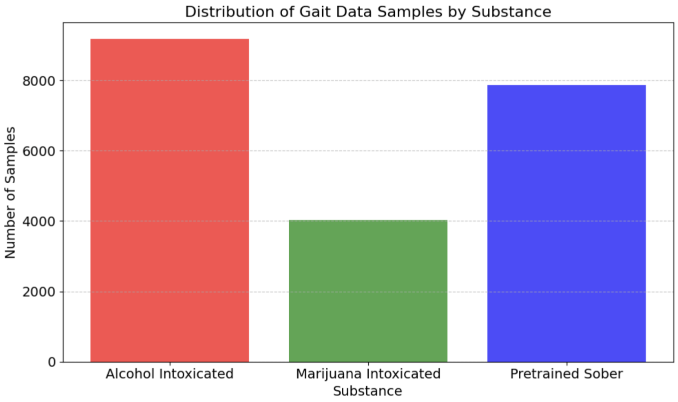

Fig S4: Distribution of samples across substances (Alcohol impaired, Marijuana impaired, Pretrained Sober).

C. Multihead Tiled CNN

Table S2 and Fig. S5 shows the Multihead Tile CNN model constructure.

TABLE S2. Multi-head Tile CNN model schema

MODEL LAYERS AND PARAMETERS

Block	Layer	Output Size
A1	Input acc_x	1@500*500
	Tiled Conv 1 (k=15, s=3,l=3)(same padding)	3@160*160
	TICA Pooling(Pooling size= 5s=2)	3@77*77
	TiledConv 2 (k=8, s=2, I=1)	3@35*35
	TICA Pooling (Pooling size = 3, s=2)	3@17*17
A2	Input acc_y	1@500*500
	Tiled Conv 1 (k=15, s=3,l=3)(same padding)	3@160*160
	TICA Pooling(Pooling size= 5s=2)	3@77*77
	TiledConv 2 (k=8, s=2, I=1)	3@35*35
	TICA Pooling (Pooling size = 3, s=2)	3@17*17
A3	Input acc_z	1@500*500
	Tiled Conv 1 (k=15, s=3,l=3)(same padding)	3@160*160
	TICA Pooling(Pooling size= 5s=2)	3@77*77
	TiledConv 2 (k=8, s=2, I=1)	3@35*35
	TICA Pooling (Pooling size = 3, s=2)	3@17*17
B1	Concatenate Layer (A1+A2+A3)	9@17*17
B2	Flatten Layer	2601

D. Tiled CNN Layer

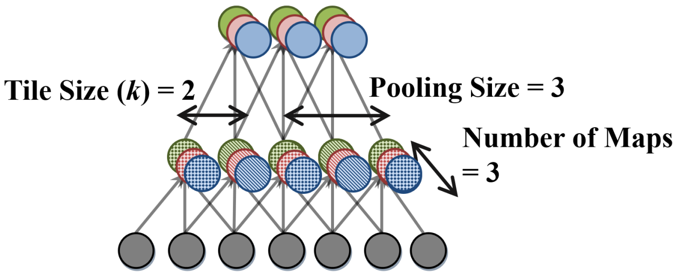

Fig. S6: Tiled CNN Layer with Pooling size 3, tile size 2, receptive fields size 3 and 3 features maps

II. RESULTS

A. Evaluation Metrics

In our study, accuracy refers to the fraction of correctly classified cases among all tested cases, expressed in equation (6). This includes correctly identifying marijuana- and alcohol-impaired individuals.

$$\text{Accuracy} = \frac{\text{TP + TN}}{\text{TP + TN + FP + FN}} \times 100 \tag{6}$$

where is TN, TP, FN, FP are True Negatives, True Positives, False Negatives, and False Positives respectively.

The F1-score is defined as the harmonic mean of Precision and Recall:

$$\text{F1-Score} = 2 * \frac{\text{Precision} \times \text{Recall}}{\text{Precision} + \text{Recall}} \tag{7}$$

where Precision and Recall are given by:

$$\text{Precision} = \frac{\text{True Positives}}{\text{True Positives} + \text{False Positives}} \tag{8}$$

$$\text{Recall} = \frac{\text{True Positives}}{\text{True Positives} + \text{False Negatives}} \tag{9}$$

The ROC-AUC (Receiver Operating Characteristic - Area Under the Curve) is the area under the ROC curve, which plots the True Positive Rate against the False Positive Rate at various thresholds.

The True Positive Rate (Recall) and False Positive Rate are defined as:

$$\text{True Positive Rate (TPR)} = \frac{\text{True Positives}}{\text{True Positives} + \text{False Negatives}} \tag{10}$$

$$\text{False Positive Rate (FPR)} = \frac{\text{False Positives}}{\text{False Positives} + \text{True Negatives}} \tag{11}$$

The ROC-AUC score is the integral of the ROC curve and provides a measure of separability between classes.

B. Baselines

To rigorously evaluate our proposed method, we compare it to several representative deep learning baseline models widely

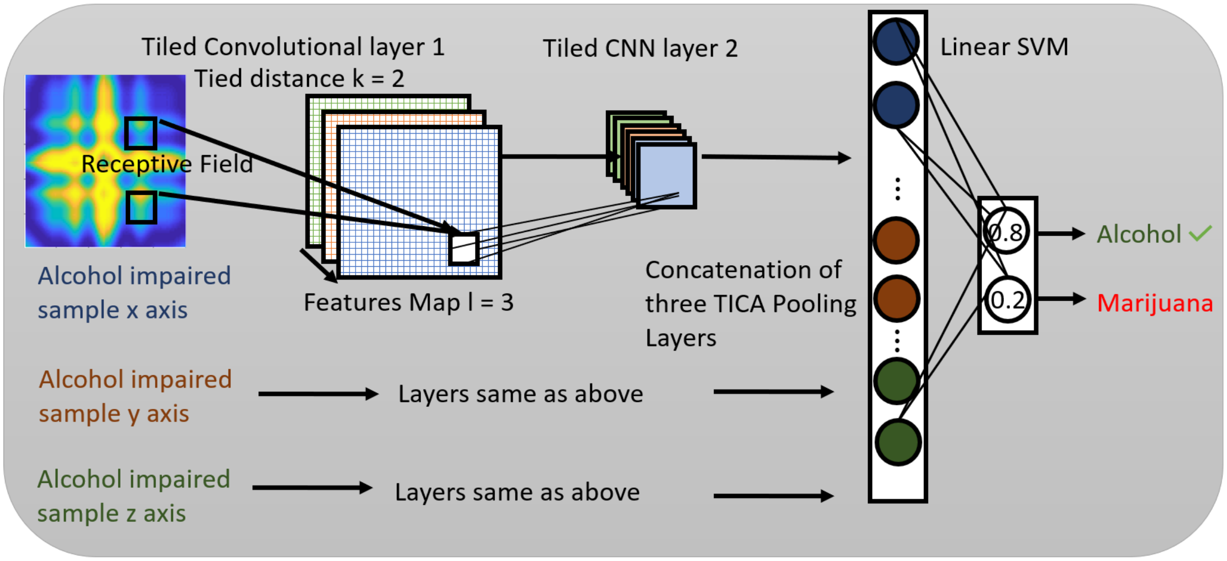

Fig. S5: Multi-head Tile CNN Model

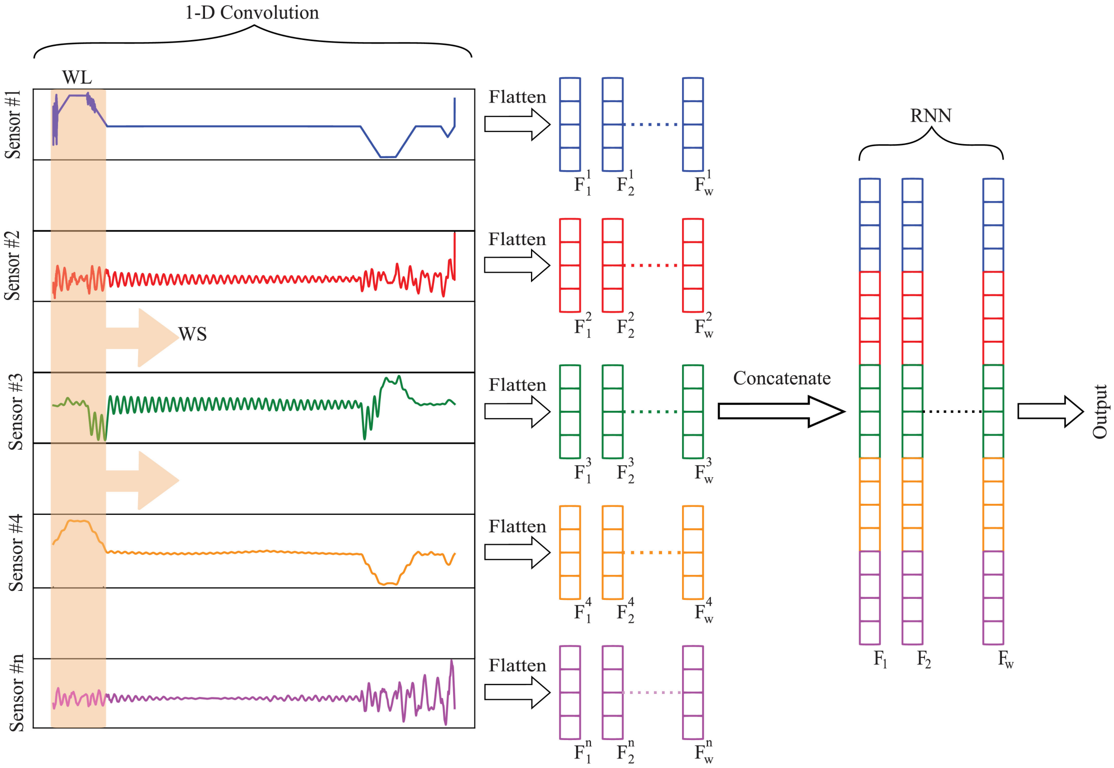

Fig S7: Multi-head LSTM Structure [5]

used in time-series and gait classification tasks, including CNNs with and without GAF, multi-head CNNs, and LSTMs.

Multi-Layer Perceptron (MLP) is a neural network that captures non-linear relationships, and is effective for basic classification tasks in biomedical signal processing. Long Short-Term Memory (LSTM) is a recurrent neural network that captures temporal dependencies, and is well-suited for analyzing sequential gait data. Multi-head CNN is a neural network architecture with multiple convolutional branches to capture both local and global features, and can capture complex spatial-temporal patterns in gait. Multi-head Long Short-Term Memory (Multi-head LSTM) [5] as shown in Fig. S7 is a neural networks architecture with multiple LSTM heads to capture varied temporal patterns, enhancing the analysis of diverse phases in gait cycles. Random Forest, is an ensemble Machine learning algorithm based on decision trees, offering robustness and interpretability, and is commonly used for high-dimensional health informatics data. It builds multiple decision trees using random subsets of the data (bootstrap sampling) and features. The final prediction is made by aggregating

the results, either through majority voting (classification) or averaging (regression). Support Vector Machine (SVM) is a binary classifier that finds optimal hyperplanes between classes, which is widely used in biomedical applications, including distinguishing subtle gait differences.

C. Experiments

Train-test curves to confirm no overfitting: Fig. S8 shows the train-test curves for the pre-trained Tile CNN with Tica pooling model on GAF-encoded images.

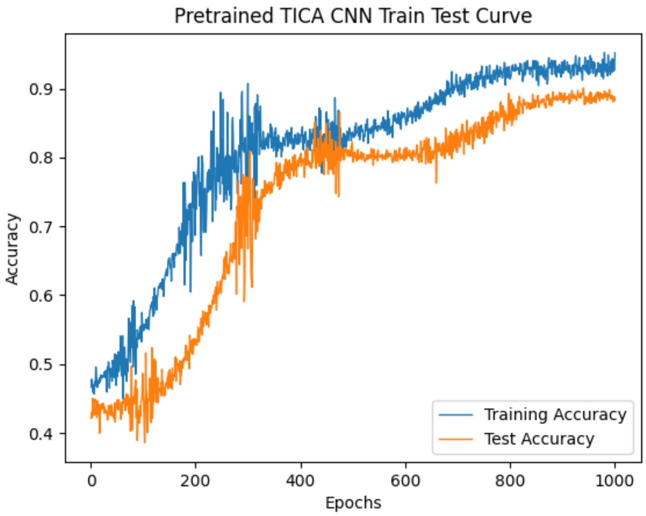

Fig. S8: Train-test curves for the pre-trained Tile CNN with Tica pooling model on GAF-encoded images, which outperformed all other models with results shown in Table IV in the main paper

III. Discussion

A. Computational Complexity Analysis

Our proposed *MariaGait* model design is efficient and computationally lightweight. Our utilization of tiled convolution significantly reduces the number of trainable parameters by enforcing weight sharing within feature maps, making it more memory-efficient than traditional CNNs. Additionally, TICA pooling enhances computational efficiency by learning orthogonal and less redundant feature sets, which reduces the complexity of the fully connected layers downstream. Unlike multi-head models or deep recurrent networks such as LSTM, the Tiled CNN with GAF encoding eliminates temporal dependencies at the model level by transforming the time-series into a spatial representation (GAF image). This enables faster inference and better parallelization on GPU hardware. Training and inference were conducted on an NVIDIA Tesla V100-SXM2 GPU (32 GB memory, CUDA 12.7), which supports high-throughput tensor operations and mixed-precision arithmetic. The GPUs 32 GB memory also enables larger batch sizes and rich spatial inputs (500×500×3) without incurring memory bottlenecks. While we do not report raw FLOPs due to system constraints, we observed that inference on a single GAF image (500×500×3) takes less than 20 ms on the Tesla V100, indicating strong suitability for real-time applications.

References

[1] Li, R., Agu, E., Sarwar, A., Grimone, K., Herman, D., Abrantes, A.M. and Stein, M.D., 2023. Fine-grained intoxicated gait classification using a bilinear CNN. IEEE sensors journal, 23(23), pp.29733-29748.

[2] Ma, Y., Fallahzadeh, R., and Ghasemzadeh, H. ”Toward robust and platform-agnostic gait analysis.” In *Proc IEEE Int'l Conf. on Wearable Implantable Body Sensor Networks (BSN)*, pages 1–6, June 2015.

[3] S. G. Liang, H. Y. Chung, K. W. Chu, Y. H. Gao, F. Y. Lau, W. I. Lai, G. C.-H. Fong, P. W.-H. Kwong, and F. M. H. Lam, “Validity and reliability of a smartphone-based gait assessment in measuring temporal gait parameters: Challenges and recommendations,” *Biosensors*, vol. 15, no. 7, Art. no. 397, 2025. [Online]. Available: https://doi.org/10.3390/bios15070397

[4] Wang, Z. and Oates, T., Encoding Time Series as Images for Visual Inspection and Classification Using Tiled Convolutional Neural Networks, in Proc. AAAI Conf. 2015.

[5] Canizo, Mikel, Triguero, Isaac, Conde, Angel, and Onieva, Enrique. ”Multi-head CNN–RNN for multi-time series anomaly detection: An industrial case study.” *Neurocomputing*, vol. 363, pp. 246–260, 2019.
